# Improved hydrogen production from pharmaceutical intermediate wastewater in an anaerobic maifanite-immobilized sludge reactor

**DOI:** 10.1039/d1ra02522h

**Published:** 2021-10-15

**Authors:** Ruina Liu, Youwei Lin, Xiaodong Ye, Jinzhao Hu, Gongdi Xu, Yongfeng Li

**Affiliations:** School of Forestry, Northeast Forestry University Harbin 150040 Heilongjiang China; School of Ecology and Environment, Hainan Tropical Ocean University SanYa 572000 Hainan China rnliu@hntou.edu.cn

## Abstract

A novel anaerobic maifanite-immobilized sludge reactor (AMSR) was employed to investigate the feasibility and performance of continuous hydrogen production for the treatment of pharmaceutical intermediate wastewater (PIW) at different organic loading rates (OLR) (from 12 to 96 g COD L^−1^ d^−1^) according to changes in the hydraulic retention time (HRT). A reactor without maifanite was also employed as a control. The results indicate that maifanite accelerates granular sludge formation and the AMSR presents more efficient and stable performance than the control in terms of the hydrogen production rate. In the AMSR, the highest hydrogen production rate of 11.2 ± 0.4 mmol L^−1^ h^−1^ was achieved at an optimum OLR of 72 g COD L^−1^ d^−1^. The main metabolic route for hydrogen production was ethanol-type fermentation, which was reflected in the relative abundance of *E. harbinense*, which was dominant for all of the OLRs. The maximum energy conversion efficiency in the dual production of hydrogen and ethanol was determined to be 24.5 kJ L^−1^ h^−1^ at an OLR of 72 g COD L^−1^ d^−1^.

## Abstract 2

An anaerobic maifanite-immobilized sludge reactor was used to investigate continuous hydrogen production for treatment of pharmaceutical intermediate wastewater at different organic loading rates according to changes in the hydraulic retention time.

## Graphical abstract

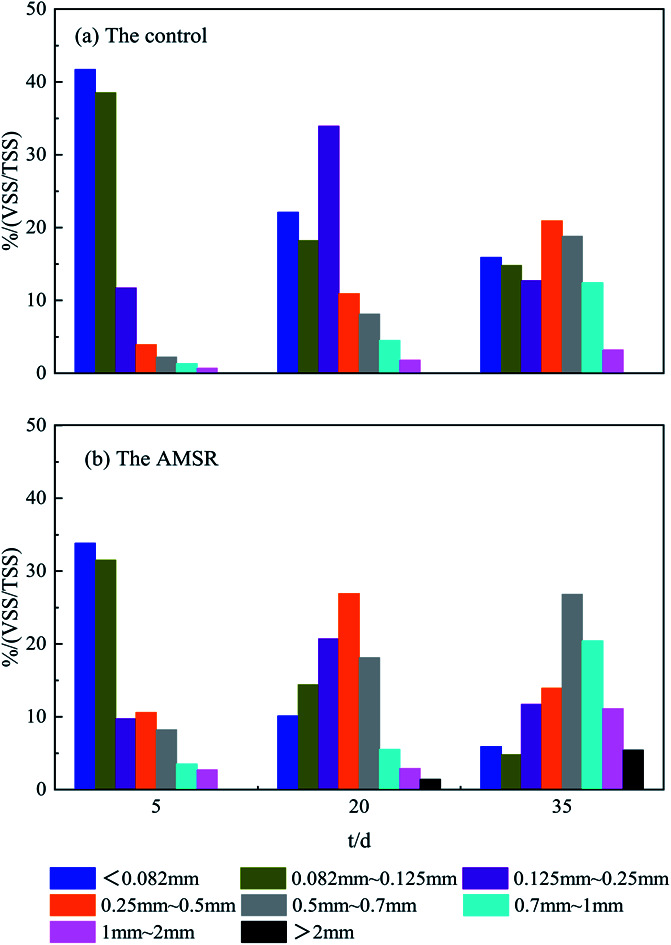

## 1. Introduction

With the gradually increasing demand for clean energy, hydrogen as a clean energy carrier generates only water as a product when combusted and has received an increasing amount of attention from researchers in recent years when faced with the issues of environmental pollution and fossil fuel depletion.^1^ Among the various methods for continuous hydrogen production, anaerobic fermentation is a promising technology, in which microorganisms degrade biodegradable matter by hydrolysis and acidogenesis under oxygen-free conditions. This anaerobic process takes place at ambient temperature and pressure with the production of harmless byproducts, so it has been proven to be less energy-consuming and more environmentally friendly compared to other technologies. Wastewater rich in a high concentration of carbohydrates can be utilized by hydrogen producing bacteria *via* hydrolysis and acidogenesis to produce hydrogen.^2^ Highly efficient hydrogen production has been so far achieved using a variety of wastewater, *e.g.*, molasses,^3^ brewery wastewater,^4^ cheese whey wastewater^5^ and rice winery,^6^ as well as palm oil mill wastewater,^7^ as demonstrated in many previous studies. It is known that most of the anaerobic fermentation processes for sequential hydrogen production are conducted in a suspended sludge system, even pure strain culture.^8,9^ A high hydrogen production rate has been achieved using a suspended sludge system in many studies,^10,11^ but the occurrence of sludge wash caused by a high organic loading rate (OLR) achieved in a low hydraulic retention time (HRT) presents a problem in the further enhancement of the hydrogen production rate. Immobilized sludge technology characterized by granular sludge is one of the technologies that can be used to reduce the phenomenon of sludge loss due to the excellent settling properties of granular sludge.^12^ A considerable number of studies have been conducted in the direction of the application of the use of an immobilized sludge system to produce hydrogen *via* anaerobic fermentation.^9,13^ So far, support materials, *e.g.*, activated carbon, calcium alginate, zeolite, and diatomite have been successfully used to promote granular sludge formation for the treatment of brewery, petroleum, and municipal wastewater, among others.^12,14,15^ Maifanite is a promising support material due to its availability and low cost. With its porous structure and large surface area, it has been widely used for heavy metal removal and dye degradation in wastewater.^16^ However, studies have been rarely carried out on the feasibility and performance of continuous hydrogen production from wastewater using a maifanite-immobilized sludge system. To our best knowledge, only Sun *et al.*^17^ have investigated biohydrogen production from traditional Chinese medicine wastewater in an anaerobic maifanite-immobilized bed reactor system, achieving a maximum hydrogen production rate of 6.40 ± 0.12 mmol L^−1^ h^−1^ at an OLR of 60.8 g COD L^−1^ d^−1^.

Pharmaceutical intermediate wastewater (PIW), produced from raw material washing, drug extraction and equipment cleaning in herbal medicine-making enterprises, largely contains carbohydrates, organic acids, glycosides, anthraquinones, lignin, alkaloids, protein, starch and their hydrolysates.^18^ It is strongly characterized by a high concentration of chemical oxygen demand (COD) and biological oxygen demand (BOD) in the range of 10 500–17 600 mg L^−1^ and 6400–13 200 mg L^−1^, respectively. PIW causes serious damage to natural water bodies and residential livelihood when directly discharged without any treatment. Nevertheless, during the treatment of PIW under certain operation conditions, the carbohydrates that exist in the PIW can be biologically converted to hydrogen by microorganisms during anaerobic fermentation. This can realize the dual environmental benefits of wastewater treatment and bioenergy recovery. To the best of our knowledge, the potential of hydrogen production from PIW *via* anaerobic fermentation was assessed was the first time by Sivaramakrishna *et al.*^19^ under different conditions, such as substrate concentration, pH and temperature in batch mode. However, in real life scenarios, PIW is continuously discharged and therefore continuous treatment is needed. Continuous hydrogen production from PIW in anaerobic processes has so far not been assessed.

Among the various parameters that influence hydrogen production, OLR is considered to be a vital parameter that can affect the enzyme activities and metabolic route of microorganisms, and subsequently influence hydrogen production.^20,21^ Usually, an optimal OLR will be decided to ensure efficient hydrogen production from an anaerobic fermentative system. Although there is considerable ambiguity about the relationship between the hydrogen production rate and OLR, it is essential to confirm the information about how the OLR influences continuous hydrogen production from PIW during anaerobic fermentation. Based on the above information, the goal of this study was to construct a novel anaerobic maifanite-immobilized sludge reactor (AMSR) to assess the performance of continuous hydrogen production from PIW *via* anaerobic fermentation at different OLRs. In addition, the microbial community structure and total energy conversion efficiency of PIW were also evaluated.

## 2. Materials and methods

### 2.1 Substrate

The PIW used as a substrate for hydrogen production was supplied by Harbin Pharmaceutical Group Co., Ltd (Harbin, China). Table 1 reveals the main chemical characteristics of the PIW. The PIW contains a high concentration of organic matter, whereas the concentrations of nitrogen and phosphorus nutrition are too low for the basic metabolism of anaerobic microorganisms. Therefore, the COD : N : P ratio of the influent was adjusted to 500 : 5 : 1 by adding a certain amount of chemicals (NH_4_Cl and KHPO_3_) to meet the microbial metabolic demands.^12^

**Table 1:** The characteristics of the PIW used in this study

Parameters	Unit	Value
Chemical oxygen demand (COD)	g L^−1^	12.21 ± 0.11
Biological oxygen demand (BOD)	g L^−1^	7.35 ± 0.23
Total suspended solid (TSS)	g L^−1^	0.56 ± 0.10
Volatile suspended solid (VSS)	g L^−1^	0.41 ± 0.06
Total nitrogen (TN)	g L^−1^	0.07 ± 0.01
Total phosphorus (TP)	g L^−1^	0.02 ± 0.01
pH	—	6.62 ± 0.28
Alkalinity	g L^−1^	0.93 ± 0.05

### 2.2 Inoculum and maifanite

The inoculum used for the startup of the reactors was raw sludge collected from the dewatering room of the Wenchang Municipal Sewage Treatment Plant (Harbin, China), which has a processing capacity of 10^5^ m^3^ d^−1^. The raw sludge was first sieved using a stainless steel colander with a diameter of 0.5 mm to remove large particles. Then, referring to the method adapted by Wang *et al.*,^22^ aeration pretreatment was carried out over a total of 30 days using sucrose as a carbon source with 2 g of COD L^−1^ in a sequential batch reactor (SBR) to suppress the metabolic activities of the hydrogen-consuming bacteria, especially the methanogenic bacteria. After sufficient enrichment, the sludge was inoculated into the reactor. The total suspended sludge (TSS) and volatile suspended sludge (VSS) were detected to be 15.62 g L^−1^ and 10.04 g L^−1^, respectively.

The maifanite was purchased from Junlian Maifanite Co., Ltd (Yixing, China). Prior to its use, the maifanite was dried in an oven at 105 °C for 1 h and sieved using a stainless steel screen with a 30 × 40 mesh. The main characteristics of the pretreated maifanite are presented in Table 2.

**Table 2:** Characteristics of the support material used in this study

Parameters	Unit	Value
Shape	—	Granules
Length	mm	5.5
Diameter	mm	2–3
Specific surface area	cm^2^ g^−1^	4.2
Point of zero charge	—	6.8
Roughness	—	14.8
Density	g cm^−3^	0.3–0.5
Moth hardness	—	1.0–1.5
Melting point	°C	1300

### 2.3 Reactor setup

Two identical reactors in this study were employed for continuous hydrogen production, one of which was packed with maifanite as a support material for biomass immobilization (packing ratio 25%), *i.e.* the AMSR and the other without maifanite used as a control for comparison of the hydrogen production performance. The reactor was composed of reinforced fiberglass, with a height of 1.0 m, an internal diameter of 20 cm, and a working volume of 30 L. A gas–solid–liquid separator was installed upside the reactor to prevent sludge loss and promote biogas release. The temperature was maintained at 37 ± 1 °C by an electric jacket and a temperature sensor was placed in the reactor for real-time detection. A pH sensor was also inserted into the reactor to monitor the system pH during anaerobic fermentation. An outlet was provided at the top of the reactor through which the generated biogas was collected using a water displacement method.

### 2.4 Experimental design

For the AMSR, the pretreated sludge was firstly inoculated into the reactors, then maifanite was added into the reactor. The two reactors were started in continuous mode at a low OLR of 2 kg COD L^−1^ d^−1^ and a HRT of 22 h, using diluted PIW as a substrate with a COD concentration of 2.04 g L^−1^. When the standard deviation of the biogas production was less than 10% at a time that was defined as the steady state, the OLR was gradually increased until 12 g COD L^−1^ d^−1^ with a constant HRT of 22 h was achieved by increasing the influent COD concentration to 12.21 g L^−1^ (Table 1). During the startup, the sludge size distribution, settling velocity (SV30) and sludge volume index (SVI) were detected regularly to observe the granulation process.

After successful startup, the OLR was gradually increased from 12 to 24, 40, 56, 72 and 96 g COD L^−1^ d^−1^ by changing the HRTs between 2.5 h and 22 h at a constant COD concentration of 12.21 g L^−1^ once a steady state was obtained at each OLR. The influent flow rate was controlled using a peristaltic pump (Model BT300, Changzhou Baist Co., Ltd., Changzhou, China) to achieve the required OLR.

### 2.5 Analytical methods

The biogas originated from the reactors was collected and monitored using a wet gas flow meter (Model LML-3, Kesion Electronics Co. Ltd., Qingdao, China). The composition of the biogas, *e.g.*, hydrogen, carbon dioxide and methane, was analyzed by gas chromatography (Model GC-2010 plus, Shimadzu, China). Nitrogen was used as the carrier gas at a flow rate of 50 mL min^−1^. The gas chromatograph was equipped with a hydrogen flame ionization detector (FID), thermal conductivity detector (TCD), flame photometric detector (FPD), electron capture detector (ECD), flame thermionic detector (FTD) and a packed column (stainless-steel 10′ × 1/8′ × 0.085′′ HayeSep D 100/120 mesh). The injector, column and detector temperatures were kept at 60, 35 and 150 °C. The composition and concentration of the soluble metabolic products, *e.g.*, ethanol, acetate acid, butyrate acid, lactic acid and propionate acid, were analyzed by liquid chromatography (Model LC-16P, Shimadzu, China) using a FID. In addition, a 2 m stainless steel column packed with a 70–80 mesh supporter was also equipped. The temperatures of the injection port, oven, and detector were 240 °C, 190 °C, and 240 °C, respectively. Nitrogen was used as a carrier gas at a flow rate of 30 mL min^−1^.

Analysis of COD, BOD, pH, TSS, VSS, TN, TP and alkalinity were conducted according to standard methods.^23^ The SV30 and SVI were measured and calculated according to the method reported by Chen *et al.*^15^ The size distribution of the granular sludge was determined as per the method proposed by Laguna *et al.*^24^ Biomass adhesion to maifanite was determined according to the methods used by Sun *et al.*^17^ The analysis of bacterial communities was performed using DNA extraction, polymerase chain reaction (PCR) and pyrosequencing, referring to the study conducted by Carosia *et al.*^25^ and Guo *et al.*^26^ Samples were added to 2 mL tubes with sterile zirconium beads of various sizes (0.3 g with a diameter of 0.1 mm and 0.1 g with a diameter of 0.5 mm; Roth). Samples were then immersed in TE (Tris-EDTA buffer solution) buffer and homogenized (3–4 min) on a shaker (Vortex Genie2) equipped with a microcentrifuge tube adapter (Mobio Laboratories; Carlsbad; USA). After shaking, 200 mL of suspension was used for DNA isolation. Microbial DNA was extracted and purified using a Power Soil DNA Isolation Kit (Mobio Laboratories; Carlsbad; USA). The quantity of the extracted DNA was checked by measuring its absorbance on a NanoVue-Plus spectrophotometer (GE Healthcare; UK). Three samples were taken for testing each time, and the data were expressed in the form of average plus deviation. The sample port was set at varying heights along the reactor.

## 3. Results and discussion

### 3.1 Granular sludge formation

**Fig. 1:**
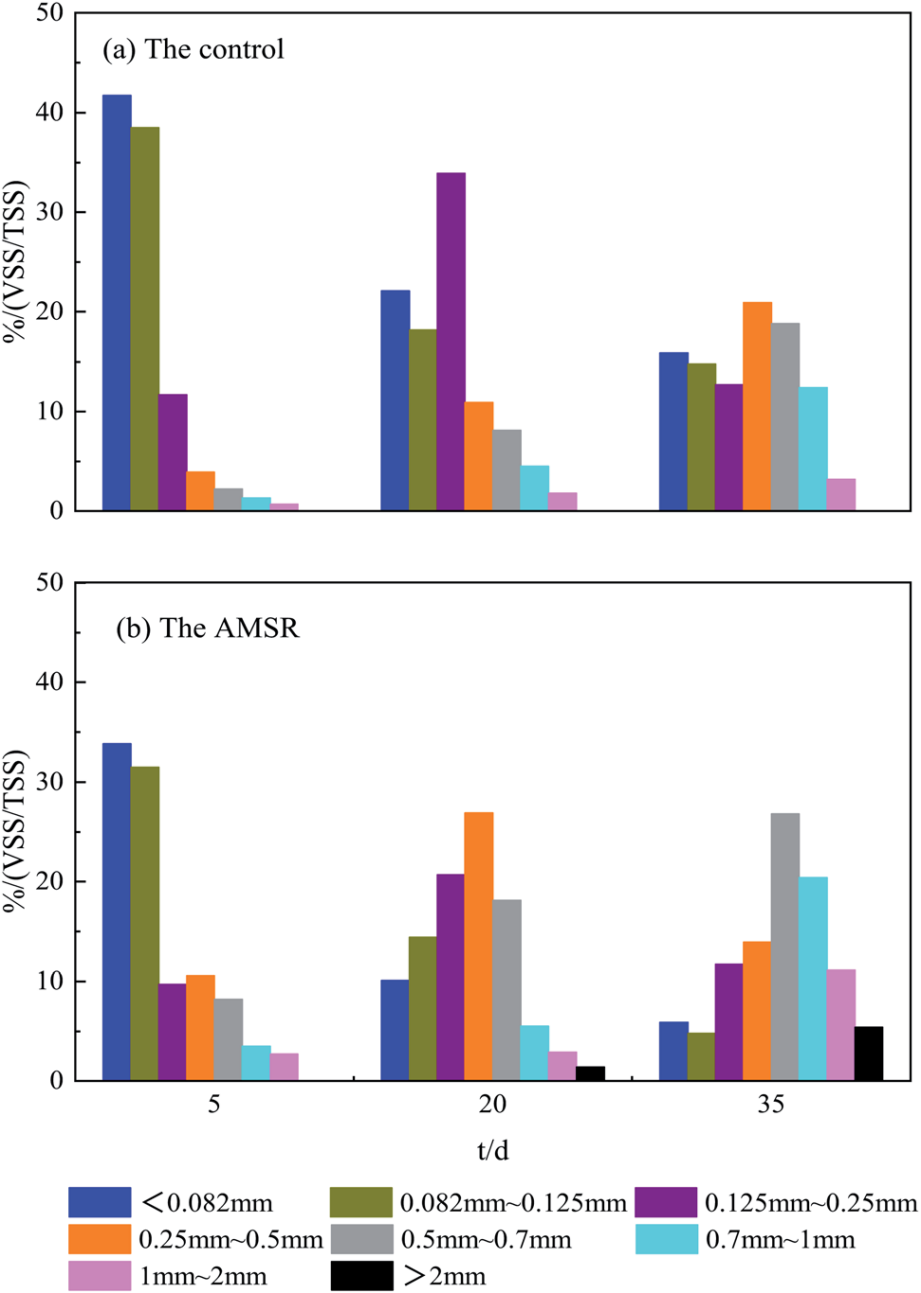
Sludge particle size distribution during the start-up processes in the control and AMSR.

The startup processes of the two reactors proceeded continuously for a total of 45 days with OLR values from 2 to 12 g COD L^−1^ d^−1^, achieved by increasing the COD concentration, and stabilized. The sludge particle size distributions during the startup processes in both the control and AMSR are shown in Fig. 1. From Fig. 1, it can be seen that the inoculum exhibits an average particle size of less than 0.082 mm, which is so-called flocculent.^1^ On day 5, the particle size of above 0.25 mm accounts for 8.1% in the control and 25.0% in the AMSR. Over days 5–20, a significant increase in the sludge particles to greater than 0.5 mm was observed for both reactors. Usually, sludge with a particle size of greater than 0.5 mm is physically defined as granular sludge during anaerobic fermentation processes in many literature studies.^27,28^ After 45 days, the granular sludge in the control and AMSR accounted for 34.4% and 63.7% respectively. The sludge particles were larger in the AMSR when compared to the control and particles larger than 2 mm were observed in the AMSR instead of the control. These observed results indicate that maifanite may provide nucleation for biomass attachment and accelerate granular sludge formation.

The date of SV30 and SVI listed in Table 3 can favor these results on the other side. The AMSR presents a lower SV30 of 18.6 ± 0.2 and SVI of 23.8 ± 0.2 mL g^−1^ compared with the control, indicating more excellent sludge settling. Previous studies have proven that a larger sludge size and rapid settling velocity enable the anaerobic reactor to reduce sludge loss and be more resistant to impact loading.^29,30^ In addition, the VSS/TSS for granular sludge in the AMSR was also less than that in the control.

**Table 3:** The characteristics of granular sludge in both reactors after successful startup

Parameter	Unit	Control	AMSR
SV30	—	26.7 ± 0.4	18.6 ± 0.2
SVI	mL g^−1^	32.5 ± 0.5	23.8 ± 0.2
VSS/TSS	—	0.75 ± 0.03	0.59 ± 0.02

### 3.2 Hydrogen production

In this study, the setup of the non-immobilized reactor as the control was only for comparison with the AMSR in terms of hydrogen production rate. Once the pretreated sludge was inoculated into the anaerobic reactor, the stable hydrogen production at an OLR of 12 g COD L^−1^ d^−1^ (COD 12.21 g L^−1^) was achieved in the AMSR, with an operation duration of 36 days. However, the control exhibited a longer startup time of 45 days to achieve a steady state. This indicates that sludge granulation using maifanite as a support material accelerates microbial colonization and acclimatization due to the high growth yield of microorganisms that favor hydrogen production.

**Fig. 2:**
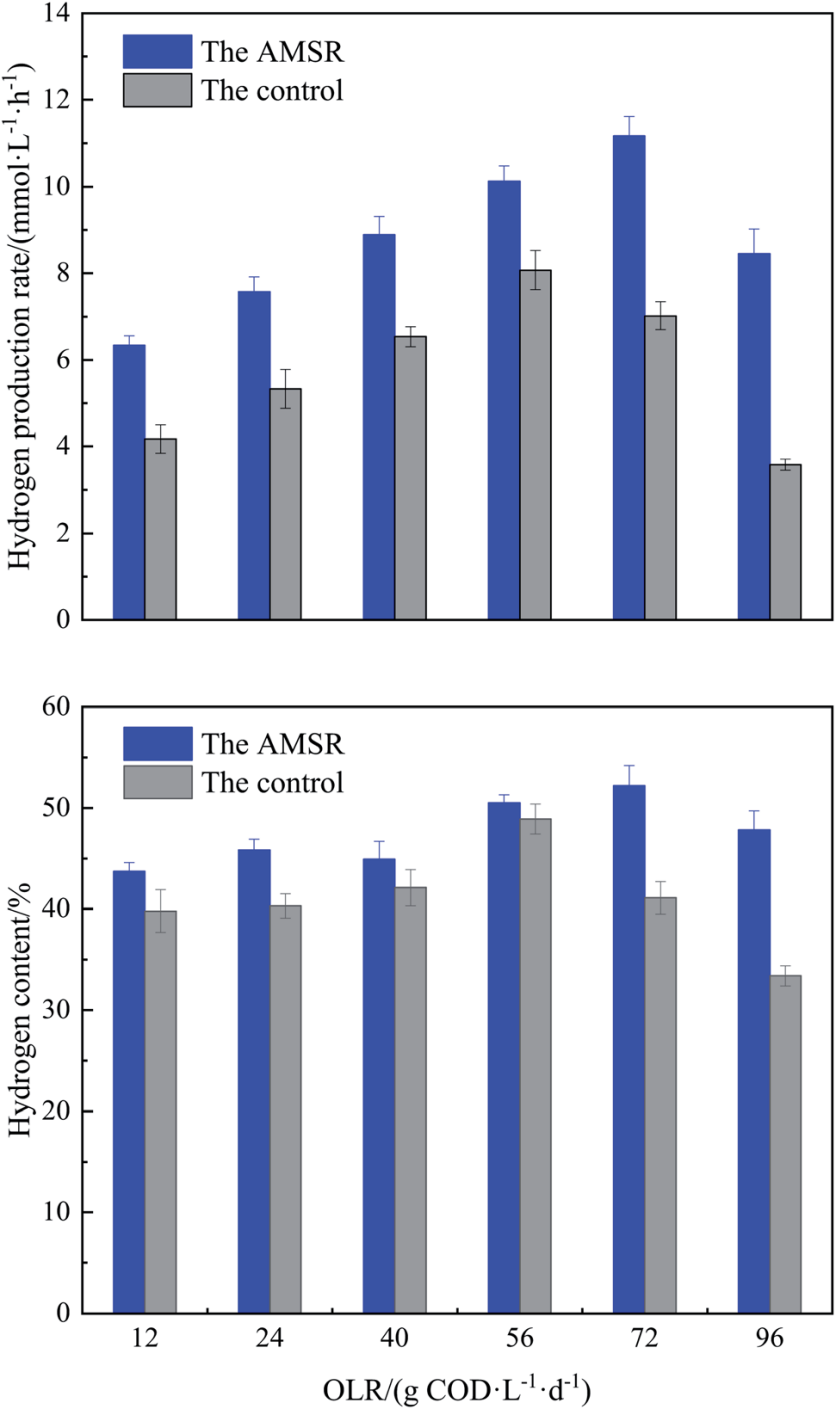
Hydrogen production rate and hydrogen content obtained by both reactors at different OLRs.

The profile of the hydrogen production rate originating from both reactors at different OLRs from 12 to 96 kg COD L^−1^ d^−1^ by decreasing the HRTs is presented in Fig. 2. It is possible to observe from this data that the AMSR is more effective and efficient compared to the control because it realizes a higher hydrogen production rate and hydrogen content during the overall operation process. For the control, the variation in the OLR ranging from 12 to 56 g COD L^−1^ d^−1^ results in a significant enhancement in the hydrogen production rate from 4.2 ± 0.2 to 8.1 ± 0.4 mmol L^−1^ h^−1^. Thereafter, a further increase in the OLR to 72 and 96 g COD L^−1^ d^−1^ causes an abrupt decrease in the hydrogen production rate to 6.9 ± 0.3 and 3.6 ± 0.2 mmol L^−1^ h^−1^, respectively. This phenomenon of decreased hydrogen production rate achieved by the control at a high OLR of above 56 kg COD L^−1^ d^−1^ may be related to the loss and washout of sludge observed in the reactor effluent due to low HRT, consequently resulting in the abatement of hydrogen producing bacteria. In addition, the inhibitory effect on the metabolic activities of hydrogen producing bacteria caused by overload OLR may also be responsible for this behavior, as described by other authors.^10^

For the AMSR, the hydrogen production rate increased gradually with the elevation in the applied OLR from 12 to 72 g COD L^−1^ d^−1^. The peak hydrogen production rate of 11.2 ± 0.4 mmol L^−1^ h^−1^ was achieved at an OLR of 72 g COD L^−1^ d^−1^, although these OLR conditions had an adverse effect on the hydrogen production of the control. The granular sludge formed in the AMSR minimizes the sludge loss and washout phenomena due to its excellent setting ability, presented in Table 2, and is more resistant to impact loading than the control. The highest hydrogen production rate resulting from the AMSR was 37.2% higher than that resulted from the control. When the OLR was further increased to 96 g COD L^−1^ d^−1^, there was an obvious decrease in the hydrogen production rate to 9.54 ± 0.57 mmol L^−1^ h^−1^ due to overload OLR, but still higher than the highest value obtained by the control under OLR conditions of 56 g COD L^−1^ d^−1^. The behavior of a decreased hydrogen production rate with exorbitant OLR has also been observed in other studies using other types of wastewater as a substrate, which reported optimal OLR ranges and that using a higher OLR exceeding the optimum conditions suppressed the hydrogen production rate. For example, Azbar *et al.*^31^ established a mesophilic continuous stirred tank reactor (CSTR) for continuous hydrogen production using cheese whey as a single carbon source under mesophilic conditions. They observed a sharp decrease in the hydrogen production rate from 9 to 6 mmol L^−1^ h^−1^ by applying an OLR range from 35 to 47 g COD L^−1^ d^−1^. Operating an anaerobic fluidized bed reactor (AFBR) to continuously produce hydrogen from molasses, Ottaviano *et al.*^5^ observed a decrease in the hydrogen production rate from 4.1 to 1.2 mmol L^−1^ h^−1^ with an increase in the OLR from 40 to 64 g COD L^−1^ d^−1^. Although more substrate is supplied by increasing the OLR, the overload substrate amount has a negative impact on hydrogen producing bacteria, thus reducing the hydrogen production rate.^19,29^

Regarding the hydrogen content present in the biogas produced from both reactors, it can be seen from Fig. 2 that the hydrogen content follows the same trend as that of the hydrogen production rate, except for the value gained at an OLR of 40 g COD L^−1^ d^−1^ in the AMSR. The AMSR exhibits range values of between 45.7 ± 0.9% and 52.2 ± 2.0%, higher than that obtained in the control under the same OLR conditions, ranging from 39.8 ± 0.7% to 48.5 ± 1.1%. The hydrogen content achieved by the AMSR was similar to other studies conducted in material-immobilized culture. Operating an induced bed reactor (IBR) for hydrogen production, Zhong *et al.*^32^ observed a highest hydrogen content of 50%. Even in a pure culture, Cappelletti *et al.*^33^ obtained an average of 56% hydrogen from anaerobic fermentation using *Thermotoga* strains. Although the OLRs had a significant effect on the hydrogen production and content, no methane was detected in the produced biogas during the entire operation process, demonstrating that the methanogenic activity was completely suppressed. Based on the above observations, the AMSR holds potential for significantly favoring anaerobic hydrogen production using PIW as a substrate. More information on the AMSR will be discussed in the subsequent section.

### 3.3 Soluble metabolic products of the AMSR

**Fig. 3:**
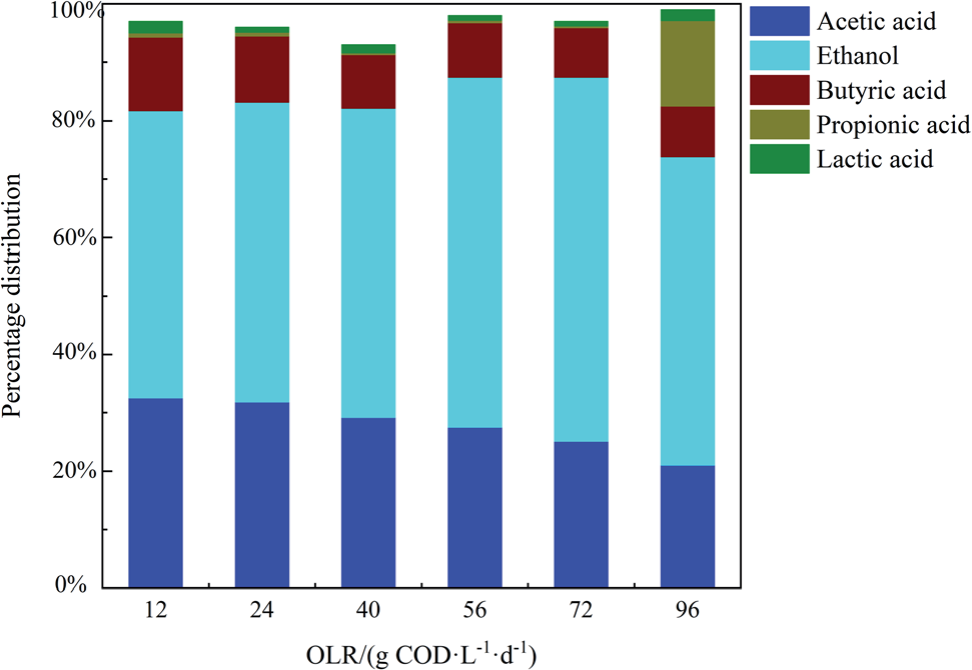
The percentage distribution of soluble metabolic products of the AMSR at different OLRs.

The production of soluble metabolic products (*i.e.* volatile fatty acids and alcohols) can take place, along with hydrogen, carbon dioxide and other byproducts during the anaerobic fermentation of organic matter.^34^ The analysis and distribution of the final metabolic products of anaerobic fermentation is of great importance because they reflect the hydrogen production performance and metabolic route of hydrogen producing bacteria.^35^ The concentrations of soluble metabolic products at different OLRs are presented in Table 4 and the distribution of each product is shown in Fig. 3. It can be seen from Table 4 that the main soluble metabolic products detected in the effluent included ethanol, acetate acid and butyrate acid for all of the OLRs and low concentrations of propionic and lactic acid were also observed. The ethanol percentage at different OLRs ranged between 49.2% and 62.3% of the total soluble metabolic products, indicating that the predominant metabolic pathway during hydrogen production was ethanol-type fermentation. Ethanol-type fermentation, in which 2 mol of hydrogen can be produced per mole of ethanol according to the reaction in eqn (1),^35^ has been confirmed to be an attractive pathway for continuous hydrogen production from various types of wastewater.

1 C_6_H_12_O_6_ + H_2_O → C_2_H_5_OH + CH_3_COOH + 2H_2_ + CO_2_

**Table 4:** Concentrations of the soluble metabolic products of the AMSR at different OLRs

OLR (g COD L^−1^ d^−1^)	Acetic acid (mg L^−1^)	Ethanol (mg L^−1^)	Butyric acid (mg L^−1^)	Propionic acid (mg L^−1^)	Lactic acid (mg L^−1^)
12	567 ± 29	893 ± 54	236 ± 11	13 ± 2	34 ± 5
24	883 ± 76	1462 ± 78	304 ± 15	17 ± 3	23 ± 3
40	821 ± 133	1899 ± 101	298 ± 22	11 ± 2	45 ± 7
56	1046 ± 98	3012 ± 203	456 ± 18	22 ± 3	39 ± 4
72	973 ± 169	3787 ± 112	417 ± 35	18 ± 3	48 ± 4
96	729 ± 77	2931 ± 178	365 ± 30	929 ± 98	98 ± 12

In this study, high ethanol concentrations were observed accompanied by high hydrogen production rates, as shown in Fig. 2. By increasing the OLR from 12 to 72 g COD L^−1^ d^−1^, there was an increase in ethanol concentration from 893 ± 54 to 3787 ± 112 mg L^−1^, reflecting an increase in the hydrogen production rate from 6.1 ± 0.3 to 11.2 ± 0.4 mmol L^−1^ h^−1^. A similar increase in the ethanol concentration by increasing the OLR from 12 to 96 g COD L^−1^ d^−1^ was also observed by Anzola-Rojas *et al.*^36^ in an anaerobic down-flow structured bed reactor (ADSBR). The pH values for all of the OLRs were in the range of 4.1–4.8 (data not shown), in keeping with the suitable pH range of 4.0–5.0 for ethanol-type fermentation, as reported in the work done by Ren *et al.*^37^ and Carosia *et al.*^25^

Notably, with a further increase in the OLR to 96 g COD L^−1^ d^−1^, the propionic acid concentration significantly increased up to a level of 829 ± 98 mg L^−1^, accounting for 14.6% of the total soluble metabolic products. As is known, propionic acid production during anaerobic fermentation is unfavorable for hydrogen production, according to the reaction shown in eqn (2),^33^ and increased propionic acid is usually accompanied by a decline in hydrogen production, as shown in Fig. 2.

2 C_6_H_12_O_6_ + 2H_2_ → 2CH_3_CH_2_COOH + 2H_2_O

Sun *et al.*^38^ observed a similar result when applying a high OLR of 84 g COD L^−1^ d^−1^ to a continuous stirred tank reactor (CSTR) for hydrogen recovery from sugary wastewater. Davila-Vazquez *et al.*^39^ also observed a maximum propionic acid concentration of 1200 mg L^−1^ with an increase in the OLR to 138.6 g COD L^−1^ d^−1^ in CSTR fed with lactose. It is well known that a higher production of nicotinamide adenine dinucleotide (NADH/NAD^+^) due to a promoted acidogenesis rate will occur once a higher OLR is applied in an anaerobic fermentative process. Therefore, the NADH/NAD^+^ ratio will be balanced through propionic acid production during which more NAD^+^ can be produced.^40,41^

### 3.4 Microbial community structure and biomass adhesion

In order to understand the influence that the applied OLR has on the hydrogen production and metabolic route in the AMSR, analysis and distribution of microbial communities from the sludge sample taken from the reactor at steady state at each OLR were performed. As shown in Table 5, the different OLRs had no obvious effect on the diversity of the microbial communities but influenced the relative abundance of each genus. Typical microbial communities were found to comprise *Ethanoligenens harbinense*, *Ethanoligenens ghanensis*, *Clostridium carboxidivorans*, *Clostridium butyricum*, *Propionibacterium cyclohexanicum*, *Sporolactobacillus inulinus* and some other species, without the presence of methanogens. These microbial communities correspond to the phyla Firmicutes, Proteobacteria, and Actinobacteria.

**Table 5:** The relative abundance of each microbial community of the AMSR detected at different OLRs

Organism affiliation	Identified (%)	Phylum	Relative abundance (%)					
			12 g COD L^−1^ d^−1^	24 g COD L^−1^ d^−1^	40 g COD L^−1^ d^−1^	56 g COD L^−1^ d^−1^	72 g COD L^−1^ d^−1^	96 g COD L^−1^ d^−1^
*Ethanoligenens harbinense*	99	Firmicutes	32.6	35.7	40.5	44.9	45.8	40.8
*Ethanoligenens ghanensis*	99	Firmicutes	10.9	10.5	8.2	9.6	11.2	8.9
*Clostridium carboxidivorans*	97	Firmicutes	20.9	18.8	20.6	15.6	17.4	12.8
*Clostridium butyricum*	99	Firmicutes	15.8	14.7	16.3	12.6	12.9	11.8
*Klebsiella pneumonia*	99	Proteobacteria	2.4	1.8	1.4	1.9	2	2.1
*Bifidobacterium longum*	97	Actinobacteria	6.5	4.8	4.9	5.5	6.1	3.9
*Propionibacterium cyclohexanicum*	95	Firmicutes	1.3	2.6	2.2	1.8	2.1	13.8
*Sporolactobacillus inulinus*	97	Firmicutes	0.9	1.4	1.7	2	1.5	5.6
*Acinetobacter calcoaceticus*	99	Proteobacteria	1.7	0.9	1.5	1	2.1	1.1

The *E. harbinense* responsible for ethanol production along with acetic acid was predominant for all of the OLRs, with a relative abundance of 32.6–45.8%. This species shows excellent high hydrogen production in anaerobic fermentative processes, so it is considered to be one of the most promising hydrogen-producing bacteria. The increased relative abundance of *E. harbinense* with an increase in the OLR from 12 to 72 g COD L^−1^ d^−1^ led to a gradual increase in ethanol production, consequently improving the hydrogen production rate. In a study conducted by Mariakakis *et al.*,^42^*E. harbinense* was also confirmed to be the main hydrogen-producing bacteria *via* ethanol-type fermentation in the ADSBR. *L. ghanensis* is another species that also participates in ethanol production along with acetic acid, as reported by Carosia *et al.*^25^ (2017) but its relative abundance was found to be within the range of 8.2–11.2%, lower than *E. harbinense*.

The second most representative species is *C. carboxidivorans*, which is one genera related to anaerobic hydrogen production during acetic acid-type fermentation, with the relative abundance of the same ranging from 12.8% to 20.9%, independent of the applied OLR. Collet *et al.*^43^ and Ratti *et al.*^44^ reported the role of *C. carboxidivorans* in hydrogen production during anaerobic fermentation. The main role of *C. butyricum* with a relative abundance in the range of 11.8–16.3% was observed in the production of butyric and acetic acid. *C. butyricum* is known to be a commonplace bacterium for hydrogen production during anaerobic fermentation dominated by a butyric acid-type pathway, as demonstrated in the literature.^45^*P. cyclohexanicum* and *S. inulinus* are responsible for the production of propionic acid and lactic acid, respectively. Anzola-Rojas *et al.*^46^ and Mariakakis *et al.*^42^ reported these species during anaerobic hydrogen production in a fixed-bed reactor and ADSBR, respectively. It is noteworthy that at an OLR of 96 g COD L^−1^ d^−1^ the relative abundance of *P. cyclohexanicum* increased from 1.3–2.2% to 13.8%, which is one of the reasons for the increase in propionate acid production shown in Table 4.

The immobilization of biofilm developed on the surface of maifanite in AMSR goes through the process of attachment, growth and detachment, of which the net result strongly influences the performance of the reactor. When the OLR increased from 12 to 72 g COD L^−1^ d^−1^, the biomass amount increased from 0.052 to 0.093 g VSS g^−1^ support. This behavior is in accordance with the hydrogen production rate. As the OLR increased to 96 g COD L^−1^ d^−1^, these values of biomass amount dropped to 0.056 g^−1^ VSS g^−1^ support, which may contribute towards the decline in the hydrogen production rate of the AMSR.

### 3.5 COD removal and pH profile

**Fig. 4:**
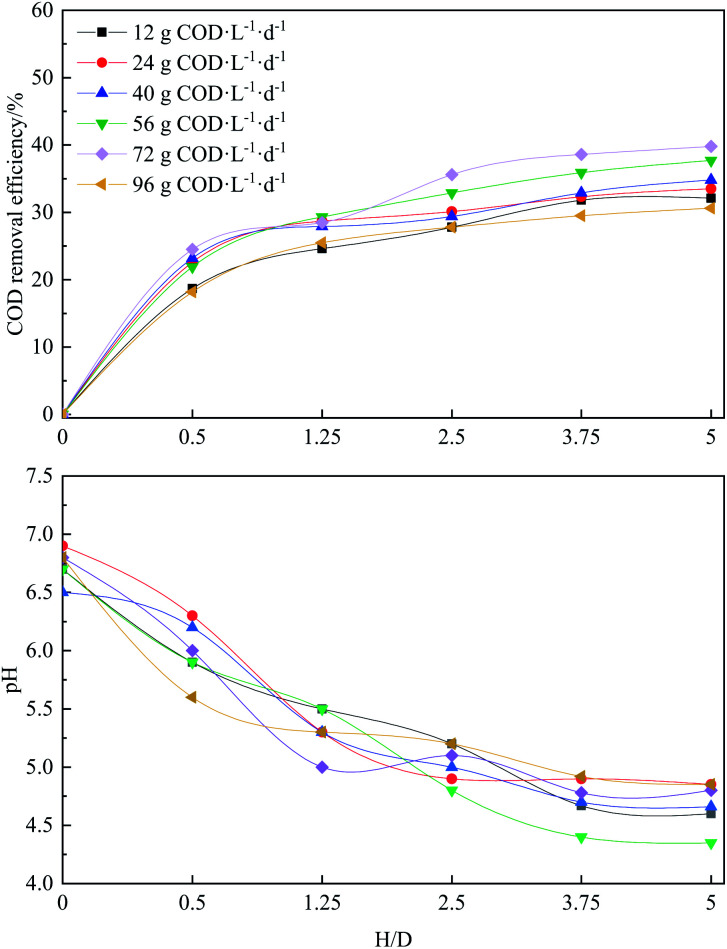
The COD removal efficiency and pH at different heights of the AMSR for all OLRs.

The analysis of the performance according to the AMSR height was carried out at each OLR. Fig. 4 shows the COD removal efficiency and pH profiles, from which it can be seen that the OLR has a significant influence on the performance at different heights of the reactor. The pH decreases over the reactor height for all of the OLRs, while the COD removal efficiency shows a downward trend. Most activities driven by microorganisms occurred below a height/diameter ratio (*H*/*D*) of 3.75 and almost no activity occurred at the remaining height. In the OLR range from 12 to 72 g COD L^−1^ d^−1^, the COD removal efficiency increases gradually from 33.5% to 39.8%, followed by a subsequent decrease to 30.6% at OLR of 96 g COD L^−1^ d^−1^. This trend is in accordance with the changes in the hydrogen production rate presented in Fig. 2 because the removed organic matter during anaerobic fermentation for hydrogen production is mainly realized by means of hydrogen release. This phenomenon could be related to the overload substrate amount resulting from a low HRT of 2.5 h, too short for microorganisms to convert the substrate, as reflected by the decreased amount of soluble metabolic product. A higher hydrogen production rate resulting from an OLR of 96 g COD L^−1^ d^−1^ than the OLRs of 12 and 24 g COD L^−1^ d^−1^ was a consequence of more substrate being supplied into the reactor. The COD removal efficiency almost reached a peak value at a *H*/*D* of 3.75, and then stabilized at the remaining height. Likewise, the pH decrease almost stopped at the same reactor height due to the maximum concentration of soluble metabolic products (data not shown).

### 3.6 Energy conversion efficiency

In this study, a maximum hydrogen production rate of 11.2 ± 0.4 mmol L^−1^ h^−1^ was obtained from PIW at an optimal OLR of 72 g COD L^−1^ d^−1^ in the AMSR. Although it is difficult to compare these results with those of other studies in terms of hydrogen production rate due to the difference in the substrate composition, operating conditions, reactor configuration and so on, they show that the conditions are interesting for recovering hydrogen from PIW using the AMSR. Han *et al.*^47^ realized a maximum hydrogen production rate of 12.5 ± 0.3 mmol L^−1^ h^−1^ at 64 g COD L^−1^ d^−1^ from molasses in a continuous mixed immobilized sludge reactor (CMISR). In other studies, using glucose as a substrate, a hydrogen production rate of 18.7 ± 0.3 mmol L^−1^ h^−1^ was achieved at an OLR of 96 g COD L^−1^ d^−1^ from an anaerobic fluidized bed reactor (AFBR) by Barros *et al.*^48^ Anzola-Rojas *et al.*^36^ operated an ADSBR using sucrose as a substrate for hydrogen generation, achieving a high production rate of 15.2 mmol L^−1^ h^−1^ with an OLR of 96 g COD L^−1^ d^−1^. Notwithstanding that the abovementioned studies reported a higher hydrogen production rate than that obtained in this study, considering that the substrates (mainly carbohydrate) used by them could be easily degraded by microorganisms, PIW containing complex components still shows excellent performance for the continuous production of hydrogen using the AMSR.

It worth highlighting that ethanol (*i.e.* liquid biofuel) can simultaneously be recovered from an anaerobic fermentation process to realize the simultaneous recovery of hydrogen and ethanol *via* ethanol-type fermentation. At an OLR of 72 g COD L^−1^ d^−1^, the ethanol production rate was also determined to have a maximum value of 15.6 mmol L^−1^ h^−1^, together with the highest hydrogen production rate of 11.2 ± 0.4 mmol L^−1^ h^−1^. Table 6 summarizes the research focused on the hydrogen and ethanol coproduction in different anaerobic reactors. Based on a method proposed by Wang *et al.*,^40^ in this study the energy conversion efficiency (considering both hydrogen and ethanol) was calculated as 24.5 kJ L^−1^ h^−1^ at an optimal OLR of 72 g COD L^−1^ d^−1^. This value obtained is comparable with other studies conducted in practical and well-proven reactors for hydrogen and ethanol coproduction. Since the reactor with maifanite as a support material allowed the formation of granular sludge, the improved energy conversion efficiency for hydrogen and ethanol coproduction when compared to the recovery of only hydrogen or ethanol could make the use of the AMSR even more promising for continuous energy recovery from wastewater. In future work, more attention will be paid towards methane production from hydrogen producing effluent rich in soluble metabolic products that are easier to catabolize by methanogenic bacteria in a maifanite-immobilized reactor.

**Table 6:** Research summary of hydrogen and ethanol coproduction in different anaerobic reactors

Reactor	Substrate	OLR (g COD L^−1^ d^−1^)	Hydrogen production (mmol L^−1^ h^−1^)	Ethanol production (mmol L^−1^ h^−1^)	Energy conversion rate^c^ (kJ h^−1^ L^−1^)	Reference
CSTR	Molasses	65	12.3	8.8	15.5	Wang *et al.*, 2013
ADSBR	Sucrose	96	15.2	14.0	23.5	Anzola-Rojas *et al.*, 2016
EGSB^a^	Molasses	120	31.7	16.3	31.3	Guo *et al.*, 2008
SCR^b^	Sucrose	1.6	1.7	0.8	1.6	Hwang *et al.*, 2004
AFBR	Glucose	96	18.8	14.8	25.4	Barros and Silva, 2012
AMSR	PIW	72	11.2	15.6	24.5	This study

a Expanded granular sludge bed.

b Semi-continuously operated reactor.

c Energy conversion efficiency = hydrogen production rate (mmol L^−1^ h^−1^) × 286 kJ mol^−1^ + ethanol production rate (mmol L^−1^ h^−1^) × 1366 kJ mol^−1^.

## 4. Conclusions

This study highlights the potential of using an AMSR in continuous hydrogen production from pharmaceutical intermediate wastewater. Maifanite provides nucleation for biomass attachment and accelerates granular sludge formation, consequently resulting in more efficient and stable performance than the control in terms of the hydrogen production rate. An increase in organic loading rate (OLR) from 12 to 72 g COD L^−1^ d^−1^ resulted in an improved hydrogen production rate to a maximum of 11.2 ± 0.4 mmol L^−1^ h^−1^, but a higher OLR of 72 g COD L^−1^ d^−1^ lowers the hydrogen production rate. The predominant metabolic pathway for hydrogen production is ethanol-type fermentation, with the ethanol percentage ranging between 49.2% and 62.3%. Corresponding, the dominant bacteria is *E. harbinense*, which is responsible for ethanol production, accounting for 32.6–45.8% for all of the OLRs. The dual recovery of hydrogen and ethanol by ethanol-type fermentation realizes a high energy conversion efficiency of 24.5 kJ L^−1^ h^−1^.

## Conflicts of interest

There are no conflicts to declare.

## Supplementary Material
